# The McGill Transgenic Rat Model of Alzheimer's Disease Displays Cognitive and Motor Impairments, Changes in Anxiety and Social Behavior, and Altered Circadian Activity

**DOI:** 10.3389/fnagi.2018.00250

**Published:** 2018-08-28

**Authors:** Tomas Petrasek, Iveta Vojtechova, Veronika Lobellova, Anna Popelikova, Martina Janikova, Hana Brozka, Pavel Houdek, Martin Sladek, Alena Sumova, Zdenka Kristofikova, Karel Vales, Ales Stuchlík

**Affiliations:** ^1^Department of Neurophysiology of Memory, Institute of Physiology of the Czech Academy of Sciences, Prague, Czechia; ^2^National Institute of Mental Health, Klecany, Czechia; ^3^First Faculty of Medicine, Charles University in Prague, Prague, Czechia; ^4^Department of Neurohumoral Regulations, Institute of Physiology of the Czech Academy of Sciences, Prague, Czechia

**Keywords:** Alzheimer's disease, transgenic, rat, cognition, social behavior, circadian system, amyloid precursor protein

## Abstract

The McGill-R-Thy1-APP transgenic rat is an animal model of the familial form of Alzheimer's disease (AD). This model mirrors several neuropathological hallmarks of the disease, including the accumulation of beta-amyloid and the formation of amyloid plaques (in homozygous animals only), neuroinflammation and the gradual deterioration of cognitive functions even prior to plaque formation, although it lacks the tauopathy observed in human victims of AD. The goal of the present study was a thorough characterization of the homozygous model with emphasis on its face validity in several domains of behavior known to be affected in AD patients, including cognitive functions, motor coordination, emotionality, sociability, and circadian activity patterns. On the behavioral level, we found normal locomotor activity in spontaneous exploration, but problems with balance and gait coordination, increased anxiety and severely impaired spatial cognition in 4–7 month old homozygous animals. The profile of social behavior and ultrasonic communication was altered in the McGill rats, without a general social withdrawal. McGill rats also exhibited changes in circadian profile, with a shorter free-running period and increased total activity during the subjective night, without signs of sleep disturbances during the inactive phase. Expression of circadian clock gene *Bmal1* was found to be increased in the parietal cortex and cerebellum, while *Nr1d1* expression was not changed. The clock-controlled gene *Prok2* expression was found to be elevated in the parietal cortex and hippocampus, which might have contributed to the observed changes in circadian phenotype. We conclude that the phenotype in the McGill rat model is not restricted to the cognitive domain, but also includes gait problems, changes in emotionality, social behavior, and circadian profiles. Our findings show that the model should be useful for the development of new therapeutic approaches targeting not only memory decline but also other symptoms decreasing the quality of life of AD patients.

## Introduction

Alzheimer's disease (AD) is a chronic neurodegenerative disease constituting a growing healthcare burden, especially in developed countries, mainly because its prevalence is bound to increase with aging of the population. Although there has been considerable research effort focused on this condition, we still lack any therapeutic tools reliably slowing its course, not to mention a cure or effective prevention.

The classical neuropathology of the disease is characterized by the presence of senile amyloid plaques, formed by overabundant amyloid-beta (Abeta) polymerizing extracellularly, intracellular neurofibrillary tangles of hyperphosphorylated tau protein, accompanied by inflammatory changes (activated microglia and astrocytes), functional and structural deterioration of synapses and neuronal degeneration. The pathology usually starts in the temporal lobes, leading to a decline of hippocampus-dependent processes, such as spatial navigation, episodic, and semantic memory. The damage then spreads into other brain regions, leading to deterioration in other functional domains as well. Early clinical manifestations are described as mild cognitive impairment (MCI), which may or may not progress into generalized dementia. The triggers of the degenerative processes are still poorly known, partly because the human brain is not easily accessible for research purposes, and partly because the patients are asymptomatic until the degeneration has been under way for a long period of time, maybe even decades (Do Carmo and Cuello, 2013; Gazova et al., 2015).

The only exception where the initial cause can be determined is the rare familial form of the disease, where the AD is linked to hereditary mutations of genes coding amyloid precursor protein (APP) (Rossor et al., 1993), or the proteins presenilin 1 and presenilin 2 linked to Abeta metabolism (Waring and Rosenberg, 2008). Identification of the mutations enabled the creation of transgenic animal models expressing abnormal APP and/or or presenilin (van Tijn et al., 2011; Sabbagh et al., 2013), featuring AD-like pathology, and allowing the use of invasive methods and observations of disease progression from the earliest stages.

The mouse was the animal of choice when the first AD models were developed and transgenic mice have elucidated some aspects of the disease, but the translation of many candidate treatments developed on mouse models to human medical practice has been problematic (Sabbagh et al., 2013). Using a wider variety of model species is therefore advisable, as it may yield results of more general validity. The laboratory rat has been advocated as a well-established experimental animal whose genetic and physiological features are closer to human, and exhibits a richer behavioral repertoire (Do Carmo and Cuello, 2013; Esquerda-Canals et al., 2017). However, the development of transgenic rat models has proven to be considerably more challenging, which has delayed their wider use.

In our study, we employed the McGill-R-Thy1-APP transgenic rat model (referred to as McGill for simplicity), expressing human APP with the Swedish and Indiana mutations responsible for familial AD in humans. The model rats start accumulating oligomeric intracellular Abeta in the cortex and the hippocampus as soon as 1 week postnatally, leading to the accumulation of extracellular Abeta and eventually the formation of neuritic plaques at the age of 6 months (in homozygous animals only), with progressive cognitive decline already apparent at the age of 3 months (prior to plaque formation). Dystrophic neurites and activated glia in the peri-plaque areas parallel the other hallmarks of human AD. Neurofibrillary tangles of hyperphosphorylated tau protein are not present, however, though this is common in transgenic AD models (Leon et al., 2010; Do Carmo and Cuello, 2013; Sabbagh et al., 2013).

Our goal was to broaden the understanding of the phenotype in the McGill rat model beyond the cognitive domain investigated in earlier studies (Leon et al., 2010; Galeano et al., 2014; Iulita et al., 2014), to determine whether it exhibits face validity (similar signs) to human AD in other respects as well. The first domain we focused on was emotionality, exploration and habituation, because increased anxiety (Porter et al., 2003) and disorientation in both novel and familiar environments (Pai and Jacobs, 2004) are common in AD patients. In the model animals, we used the open field, elevated plus maze and light-dark tests to assess those aspects of behavior. AD patients often experience gait and balance problems, often leading to serious injuries (Visser, 1983; Rossor et al., 1993; Suttanon et al., 2012). We included the beam walking test, to probe locomotor coordination in the rat model. Cognitive function impairments are the hallmark of human AD. We performed a task focused on delay-dependent forgetting in the Morris water maze, and also the passive and active place avoidance tasks in the carousel maze, a paradigm known to be sensitive to AD-related impairments (Petrasek et al., 2016), but never before applied to this particular model. As those tasks tap different aspects of spatial cognition, they provide complementary information, and are well-suited for inclusion in testing batteries, as previous experience with one of them does not affect the performance in the other, as shown by Vojtechova et al. (2016). We also included a test of working memory in the Y-maze and recognition memory in the novel object recognition and novel object location tasks. General social withdrawal or a failure to recognize familiar individuals are common in human AD (Tak and Hong, 2014). Therefore, we looked into the social behavior of the animals, evaluating their social exploration of a conspecific and social recognition memory.

In addition, we also characterized the circadian system of the model. Disturbances of the circadian system are common to neurodegenerative diseases such as AD and often precede other signs and symptoms (Kondratova and Kondratov, 2012; Hood and Amir, 2017), while some pathophysiological aspects of the disease, such as Abeta secretion, exhibit circadian variation (Bayer and Wirths, 2014). The circadian aspects of behavior and physiology are governed by the central pacemaker in the suprachiasmatic nuclei of the hypothalamus (SCN). The cells of the SCN form a complex network of mutually synchronized cellular oscillators that depends on communication via neurotransmitters and provides both a robust circadian signal for other body clocks as well as flexible entrainment to external light-dark cycle via a direct retinal connection (Welsh et al., 2010). Both the SCN and the peripheral clocks in other parts of the brain and almost all non-neuronal tissues are dependent on the rhythmic expression of clock genes such as *Per1/2, Cry1/2, Nr1d1/2*, and *Bmal1/2* (Mohawk et al., 2012), which form transcriptional-translational feedback loops and regulate local tissue-specific clock-controlled genes.

To examine the circadian system of the McGill rats we exposed the rats to a battery of external lighting conditions in order to ascertain its functionality, which we hypothesized to be compromised by Abeta accumulation. We analyzed the locomotor activity patterns, and assessed the functional state of the circadian clocks in selected brain areas by detecting the expression of the clock genes *Nr1d1* (also called *Rev-Erb*α) and *Bmal1* (also called *Arntl*) in the hippocampus, parietal cortex and cerebellum. Additionally, we analyzed the expression of the clock-controlled gene *Prok2* (coding neuropeptide prokineticin 2) that is involved in regulation of the sleep/wake cycle, activity level and neuroinflammatory state (Cheng et al., 2002) and whose mRNA is upregulated by various stressors including amyloidosis (Severini et al., 2015).

## Materials and methods

### Animals

McGill-R-Thy1-APP transgenic rats (McGill) carry a single transgene (one copy per haploid genome), expressing human APP_751_ carrying the Swedish and Indiana mutations under the control of the murine Thy1.2 promoter, on the genetic background of HsdBrl:WH Wistar rats. The transgenic product is expressed primarily by telencephalic neurons and is not detectable in the cerebellum or peripheral tissues. The genetic manipulation and initial phenotyping of the McGill-R-Thy1-APP model is described in Leon et al. (2010).

In the present study, we used 10 male homozygous transgenic McGill-R-Thy1-APP rats (McGill), coming from three different litters, and 10 age-matched non-littermate wild type controls (WT), also from three litters. The rats were obtained from PsychoGenics Inc., 765 Old Saw Mill River Road, Tarrytown, NY 10591. The same animals were used for all the experiments described (except for the analysis of the daily profile of *Nr1d1* and *Bmal1* expression, described in section Clock Gene Expression in the Brain). The animals were housed in an air-conditioned animal room with 12-h light/12-h dark cycle unless otherwise stated. Two or three littermates always shared the same transparent box (44 × 28 × 23 cm), with food and water available *ad libitum*, with the exception of experiments requiring food or social deprivation.

The animals were allowed a 25-day acclimation period prior to the testing. All behavioral experiments were conducted during the light phase of the day, unless otherwise noted. The animals from different groups were always tested in alternating order, to minimize the possibility of circadian changes in behavior or external disturbances producing false positive results. All the apparatuses were thoroughly cleaned by water after each animal, to erase or smear any scent marks.

When the observations were finished, the rats were sacrificed at the age of 10 months and their brains analyzed. The timeline of experimental procedures can be found in Table 1.

**Table 1:** Timeline of the experiments.

**Procedure**	**Age (months)**
Arrival to animal facility	3.6
EPM1; EPM2	4.4
OF1; OF2; beam walking	4.5
EPM3	4.6
OF3	4.7
MWM	4.7–5.1
Y-maze1; light-dark test	5.1
Carousel maze testing	5.4–5.9
Y-maze2	6.0
Social recognition memory	6.1–6.3
NOR; NOL	6.7
Social interaction	6.8
Y-maze3	8.1
Chronobiological assessment	8.2–10.0
End of experiments	10.4

All experiments were approved by the local Committee for Animal Protection and complied with the Animal Protection Act of the Czech Republic, EU directive (2010/63/EC).

### Elevated plus maze (EPM)

The EPM is a standard test of anxiety and spontaneous exploration, where the animals choose between exploring the closed arms, perceived as safer, or the more exposed open arms of the apparatus. We used the same apparatus described in Vojtechova et al. (2018).

Three 5-min testing sessions took place, with the initial exposure to the apparatus repeated after 1 day and again after 7 days, to assess possible differences in habituation.

The behavior of the animals was recorded by a web camera placed above the apparatus. Locomotor activity (**total distance**) of the animals was analyzed offline by means of the digital tracking software Viewer 3 (Bioobserve GmbH). **Total time spent in each compartment** was evaluated by an investigator using EthoWatcher software (Crispim Junior et al., 2012).

### Open field test (OF)

The animals were placed in the middle of a brightly lit square-shaped arena (70 × 70 cm) with dark floor and walls. Each experimental session lasted 10 min, and the movement of the animals was recorded by a camera placed above the apparatus.

Three testing sessions took place, with the initial exposure to the apparatus repeated after 1 day and again after 7 days, to assess possible differences in habituation. We hypothesized that lower habituation in the McGill group might indicate failure to recognize an already-visited environment.

The trajectory of the animals was analyzed using Viewer 3. **Total path length** and **the percentage of time spent in the central 50% of the arena** were assessed. The videos were also scored by a human observer using EthoWatcher and the most distinctive behaviors (**rearing, grooming, freezing, defecation**) noted.

### Beam walking

This task assesses sensorimotor coordination (Goldstein, 1993). We used a 2 m long wooden beam, stretched between a blinded end and a home cage. The beam was placed so that the animals walked either the wide (5 cm) or narrow (2.2 cm) side, and the distance between the blinded end and the goal varied (see Table 2 for design of the experiment).

**Table 2:** Beam walking test design.

**Trial**	**1**	**2**	**3**	**4**	**5**	**6**	**7**	**8**	**9**
Width	5 cm	5 cm	5 cm	5 cm	2.2 cm	2.2 cm	2.2 cm	2.2 cm	2.2 cm
Length	0.5 m	1 m	2 m	2 m	1 m	2 m	2 m	2 m	2 m

The performance of the animals was watched by two observers, each viewing the animal from one side, measuring the **time** needed for the animal to traverse the beam (the trial was terminated if the animal didn't reach the end in 1 min), recording **the number of foot slips** (when one paw of the animal slipped from the upper surface of the beam), **near-falls** (severe loss of balance not leading to actual falls), and actual **falls**. We set the value of 10 as the arbitrary maximum number of foot slips, and used it for all animals unable to walk properly on the top surface of the beam.

### Y-maze

In this test, the animals were placed into the middle of a three-armed radial maze, with each arm 39 cm long and 10 cm wide; and were left free to explore the empty apparatus for 8 min. We conducted three sessions to observe whether individual or group patterns of behavior remain stable in the long-term. The experimental protocol and analysis were similar to the experiment done by Galeano et al. (2014) in hemizygous McGill animals, to enable easier comparison of the results.

A visit to an arm was counted by the experimenter if the rat placed all 4 paws into the arm. **Spontaneous alternation** was measured as the ratio of actual triads (three different arms entered in three subsequent visits) to potential triads (theoretical maximum performance). It is assumed that rats prefer to alternate between the visited arms if they remember past choices; therefore, the alternation is a measure of working memory. From the order of visits, the number of left (L) and right (R) turns was derived, and **the laterality index** indicating side preference was calculated as (L-R)/(L+R).

### Morris water maze (MWM)

The Morris water maze is a test of precise place navigation and memory (Morris, 1981, reviewed in D'Hooge and De Deyn, 2001). We used the visible platform version to test the general ability of animals to solve the task, and the delayed-matching-to-place protocol to test delay-dependent working memory retention. The procedures and the apparatus were almost identical to our previous work (Petrasek et al., 2014).

The MWM was filled with water at a temperature of 21 ± 2°C, which was darkened by a small amount of non-toxic paint (SwingColor, Bauhaus, Czech Republic). If the animals failed to reach the hidden platform within 1 min, they were led to it by the experimenter. The animal position was monitored by an overhead camera connected to a digital tracking system and data acquisition program (Tracker, Biosignal Group, USA).

In the **visible platform testing** (two daily sessions) the platform was raised above the surface and marked by a white rim, and an additional cue (made from two CDs) was suspended ~20 cm above the platform. The animals underwent eight trials per session in 15-min intervals, always being released from one starting location, while the platform position was pseudorandomly changed between trials.

The **delayed-matching-to-place** task (first described by Steele and Morris, 1999; O'Carroll et al., 2006) is a test of one-trial spatial learning, permitting an analysis of spatial working memory and engram persistence.

The platform position was pseudorandomly changed each day (each position being unique), but remained stable for all trials during a particular daily session. The animals had four trials per day, beginning at pseudorandomly chosen starting points. The first trial should essentially be a random search for the platform, while on subsequent trials the animals could use the knowledge of the platform position to find it more quickly. The delay between the first and the second trial (inter-trial interval, ITI) was either short (15 s) or long (2 h). The ITIs between the second, third, and fourth trials were always 15 s to maintain a win-stay intra-session strategy. The testing took 8 days, but only the data from days 3 to 8, when the animals were already familiar with the task rules, were used for statistical analysis.

Performance in the MWM was measured by **escape latency** to reach the platform. **Savings** between the first and second trial were used as an indicator of one-trial learning.

We also included **swimming speed** as a measure of locomotor skills, and **thigmotaxis** (the percentage of time spent in the peripheral part of the pool) as a measure of anxiety and searching strategy.

### Carousel maze testing battery

The carousel maze (originally described by Bureš et al., 1997; Fenton et al., 1998; reviewed in Stuchlík et al., 2013, 2014) consists of a circular metallic arena, which can be rotated. A 60-degree wide to-be-avoided sector (directly imperceptible to the animals) can be defined in the tracking software, and its position must be learned relative to a relevant set of spatial cues. For a detailed description of the apparatus, see Petrasek et al. (2014, 2016).

The trajectory of the rats was analyzed offline using Carousel Maze Manager 4.0 (Bahník, 2014). Ultrasonic vocalizations were recorded using an Ultramic 250k microphone (Dodotronic, Italy) and Audacity 2.1.0 software. The recordings were then assessed visually for the presence or absence of vocalizations.

Each session lasted 20 min (except rotation habituation which lasted 10 min). The design of the testing battery is described in Table 3.

**Table 3:** Carousel maze design.

**Day**	**1**	**2**	**3**	**4**	**5**	**6**	**7**	**8**	**9**	**10**	**11**	**12**	**13**	**14**
Task	Spontaneous foraging			Passive place avoidance				Rotation habituation	Active place avoidance					
Description	Stable arena, no foot shocks			Stable arena, stable sector				Rotating arena, no foot shocks (10 min only)	Rotating arena, stable room-frame sector					

The rats were food-deprived to 90 ± 5% of their normal weight and foraged for groats falling (~3 groats per minute) onto the arena from an overhead feeder in all carousel maze sessions, to motivate them to explore the arena (also see Stuchlík et al., 2013).

At the beginning, the rats had 3 days of **spontaneous foraging** on a stable arena, to familiarize themselves with the experimental apparatus and collecting the groats. In the **passive place avoidance**, a to-be-avoided sector was defined on the arena surface, which the rats had to avoid. As both the arena and the sector were stable, the task was not particularly difficult and could be solved by merely suppressing locomotor activity and staying anywhere outside the sector.

On Day 8, the shock was switched off, and the arena was rotated at the speed of 1 rotation per minute (**rotation habituation**). This session was included mainly for the animals to get used to arena rotation (esp. the noise made by the motor driving the arena).

Since Day 9, the animals learned to solve the **active place avoidance** task (also known as active allothetic place avoidance). The arena rotated while the sector remained fixed in the reference frame of the room. The rats had to move actively away from the sector in the direction opposite to arena rotation in order not to be passively transported into the sector. For successful avoidance, the animal had to distinguish the distant room-frame cues that could be used to locate and avoid the sector from the irrelevant arena-frame cues (i.e., scent marks) which were useless and misleading. The active place avoidance has lower demands on precise spatial navigation than the MWM (the sector is quite large and the animals are not required to locate it precisely), but on the other hand, selection of the correct spatial cues and achievement of the correct behavioral strategy requires the segregation of spatial frames, a skill that is considered the equivalent of human cognitive coordination (Wesierska et al., 2005). Maintaining two spatial representations at once and choosing the relevant one is a function that is especially sensitive to hippocampal damage (Kubík and Fenton, 2005; Stuchlík et al., 2013).

In the carousel maze, **total distance** traveled within a session served as a measure of locomotor activity in all tasks. **Mean distance from the center** indicated the thigmotactic behavior of the animals.

In the passive and active place avoidance tasks, we assessed the **number of entrances** into the to-be-avoided sector as a measure of avoidance, and the **number of shocks per entrance** (skill learning index), calculated as (number of entrances + 1)/(number of shocks + 1). This parameter is sensitive to the behavior of the rat when it enters the sector, namely whether it responds to the first shock with an immediate escape, or fails to leave the sector, receiving additional shocks.

### Light-dark test

In this task, the rats explored a box (45 × 45 cm) divided into two compartments of equal size, connected by a small, open door. The dark compartment was enclosed by black, opaque walls and ceiling, while the light compartment had transparent walls and was open from the top. The whole apparatus was placed inside a white box, and brightly (but indirectly) lit.

The rats were always inserted into the light compartment. The experimental session lasted 5 min and was recorded by a digital camera placed above the maze. The behavior of the animals was scored by an observer using EthoWatcher. An **entrance** into a compartment was counted when the rat entered a compartment with all 4 paws. **Risk assessment** behavior was defined as looking into the light compartment without actually entering it.

### Social recognition task

To investigate cognitive domains other than spatial memory, we included a test of social recognition memory in a resident-intruder paradigm. The test is based on the assumption that adult rats would investigate unfamiliar conspecifics more than familiar ones, unless their social recognition memory is impaired, in which case they fail to recognize already-encountered individuals. The testing protocol was similar to Lemaire (2003), using juvenile Wistar rats (23–29 days old) as stimulus animals. Juvenile males are old enough to be recognized by the adult animals as distinct individuals, while usually not taken as territorial rivals and therefore not eliciting aggressive responses. We hypothesized that McGill rats would exhibit decreased social memory and possibly also a generalized deficit of social behavior.

The McGill and control rats were socially isolated in standard cages (43 × 28 × 23 cm) in the experimental room for 24 h to ensure their resident status, and food- and water deprived for 3 h before the first experimental session. The stimulus animals were isolated in the same room, in clean boxes without food and water for 3 h before contact with the adults. The experiment always began with **an acquisition session**. A cage with the tested adult rat was placed under a camera and a juvenile intruder was introduced into the cage. The behavior of the pair was recorded for 5 min, and then the rats were separated again. After a 30-min delay, **a retrieval session** was conducted in the same way as the acquisition session. In the retrieval session, the adult was exposed either to the same juvenile (familiar) or a completely novel one (unfamiliar). The adult rats were tested again the next day using essentially the same procedure, except that the adults that received an unfamiliar juvenile the day before interacted with a familiar one and the other way round. No adult interacted with any of the juveniles experienced the day before.

The video recordings were analyzed manually, using the EthoWatcher software. The following behaviors of the adult rats were noted: **non-anogenital investigation** (sniffing or touching the head or the back of the juvenile); **anogenital investigation** (sniffing or licking of the anogenital region of the juvenile); **following** (chasing the juvenile or following its movements); **pinning** (deliberate crawling over the juvenile and/or holding it underneath); **social evasion** (withdrawal from social interaction by escape or kicking) and **passive contact** (contact initiated by the juvenile, including touching, crawling over or under, or following the adult, without the adult investigating the juvenile).

### Social interaction with an unfamiliar adult

Social interaction took place in the same environment as the OF. The arena was thoroughly cleaned by ethanol and water prior to every session. The animals were not isolated before the test. Two rats of the same genotype, but coming from different cages (and therefore unfamiliar to each other) were placed into the OF together, and left undisturbed for 10 min. Their behavior was recorded by an overhead camera and scored by an observer blinded to the genotype, using the BORIS software (Friard and Gamba, 2016). The behaviors noted were **non-anogenital social contact** (intentional touching or sniffing each other outside the anogenital area), **anogenital exploration**, **following**, and **self-grooming**.

Ultrasonic vocalizations of the rats were monitored by an ultrasonic microphone (Ultramic 250K; Dodotronic, Italy) at a sampling frequency 250 kHz, and scored manually using the software Audacity 2.1.0. While we refer to the classification proposed by Wright et al. (2010) in the call subtype description below, in practice we used more general categories to avoid ambiguities.

The three most abundant and distinctive call categories were **simple high-frequency vocalizations** (calls without major steps or trills, corresponding to types 1–4 and 13 in the classification by Wright et al., 2010), **trills** (high-frequency calls with rapid, periodic oscillations over a wide range of frequencies, corresponding to types 10–12 in Wright et al.), and **stepped and composite vocalizations** (calls containing notable steps in frequency, or a combination with trills, corresponding to types 6–9 and 14 in the Wright classification). Examples of the calls can be seen in **Figure 10**. Because vocalizations cannot be attributed to individual animals, pairs were taken as a single unit for statistical purposes.

### Novel object location/recognition (NOL/NOR) task

The test consisted of three parts: habituation, novel object location (NOL—training, test) and novel object recognition (NOR—training, test). On the first day, an animal was put into the black square arena (70 × 70 cm, the same as used for OF testing) for 5 min to allow **habituation** to the environment. The following day, two identical objects (glass jars full of pebbles) were added into the arena to opposite corners, 15 cm from the walls and the rat spent there 5 min exploring the objects (**NOL training phase**). After a 1 h retention interval, one object was shifted by 20 cm and the animal was allowed to explore the objects for 3 min (**NOL test phase**). On day 3, the animals explored the same identical objects in the same location as the previous day for 5 min (**NOR training phase**). After the 1 h retention interval, one object was changed to a novel one (glass cuboid container of a comparable size) and the rat explored both objects for 3 min (**NOR test phase**). In both tasks, object relocation and change were counterbalanced in both groups of animals to minimize the effect of any eventual object or place preference unrelated to the experimental stimulus. The behavior of the animals was analyzed by a human observer using the EthoWatcher software, who noted **object exploration** (sniffing or closely examining the object) and **object climbing**.

The following parameters were computed:

$$\begin{array}{l} \text{discrimination index}={\text{T}}_{\text{n}}-{\text{T}}_{\text{f}}\\ \text{discrimination ratio}=\left({\text{T}}_{\text{n}}-{\text{T}}_{\text{f}}\right)/\left({\text{T}}_{\text{n}}+{\text{T}}_{\text{f}}\right) \end{array}$$

where T_f_ is the total time spent exploring the familiar object, while T_n_ is time spent by exploration of the relocated (in NOL) or novel (in NOR) object.

### Chronobiological assessments

#### Experimental protocol to study circadian behavior

Eight-month-old rats were housed individually with free access to food and water and exposed to several different consecutive light regimes, starting with 12 h of light and 12 h of darkness (LD) for 17 days, then constant darkness (DD) for 16 days, LD12:12 again for 10 days, and finally constant light (LL) for 13 days. Animals were continuously monitored for spontaneous locomotor activity.

#### Monitoring of circadian locomotor activity

The locomotor activity was monitored in cages equipped with infrared movement detectors that were attached above the center of the cage top using a circadian activity monitoring system (Dr. H.M. Cooper, INSERM, France). The movement was measured and binned every minute and double-plotted actograms were generated to evaluate the activity. The resulting data were analyzed using the ClockLab toolbox (Actimetrics, USA) and Actiwatch Activity & Sleep Analysis V 5.42 software (Cambridge Neurotechnology Ltd, UK). The analyzed parameters were total activity (average counts/day during the specified interval), the activity/rest ratio, the period and power of the rhythm, the percentage of time spent in an inactive state (as a correlate of sleep) and the fragmentation index (i.e., the percentage of restlessness during inactivity).

#### RNA isolation and real-time RT qPCR

The rats were sacrificed by cervical dislocation, and samples of the hippocampus, parietal cortex and cerebellum were removed, frozen on dry ice and kept at −80°C until processing. RNA purification and RT qPCR detection of mRNA were performed as described previously (Sládek et al., 2007a; Polidarová et al., 2017). Briefly, total RNA was extracted by rotor-stator homogenization and subsequently purified using the GeneElute Total Mammal RNA kit (Sigma, USA). RNA concentrations were determined by spectrophotometry at 260 nm, and the RNA quality was assessed by electrophoresis on a 1.5% agarose gel. Moreover, the integrity of randomly selected samples of total RNA was tested using an Agilent 2100 Bioanalyzer (Agilent Technologies, USA). One microgram of total RNA was reverse transcribed using a High Capacity cDNA RT kit (ThermoFisher, USA). Diluted cDNA was then amplified on a LightCycler480 (Roche, Basel, Switzerland) using SYBR Select qPCR Master Mix (ThermoFisher, USA) with the corresponding primers for *Nr1d1, Bmal1, B2m*, and *Gapdh* (see Sládek et al., 2007a and Polidarová et al., 2017, for sequences), or using TaqMan qPCR Master Mix (Solis-BioDyne, Estonia) with *Prok2* (Rn01421308_m1, FAM) and *Gapdh* (endogenous control, VIC, primer limited) TaqMan probes (ThermoFisher, USA) in duplex. Relative quantification was achieved by the ΔΔCt method (Livak and Schmittgen, 2001) with *B2m* and *Gapdh* as controls. Both reference genes have been previously used for normalization (Sládek et al., 2007a,b, 2012; Polidarová et al., 2011, 2013) and their expression was stable throughout the 24 h and across the tested tissues.

### Statistical evaluation

Behavioral data were analyzed by analysis of variance (ANOVA) with repeated measurements, or *t*-tests when applicable.

Circadian period was analyzed by an unpaired two-tailed *t*-test with Welch's correction, while the power of period, activity/rest ratio (both representing the amplitude of the locomotor rhythm) and total activity were analyzed by the unpaired two-tailed *t*-test. Sleep inactivity and sleep fragmentation index were analyzed separately for active and inactive phase and the differences between WT and McGill rats were tested by one-way ANOVA with Šidák's multiple comparisons. Gene expression levels were analyzed by two-way ANOVA with Holm-Šidák's multiple comparisons. The 24 h gene expression profiles were analyzed by one-way ANOVA and the cosinor test to detect rhythmicity as described previously (Polidarová et al., 2013). *P* < 0.05 (multiplicity adjusted in case of ANOVA) was required for significance. All of the statistical calculations were performed with Prism 7 software (GraphPad, USA).

## Results

### Elevated plus maze

**Figure 1:**
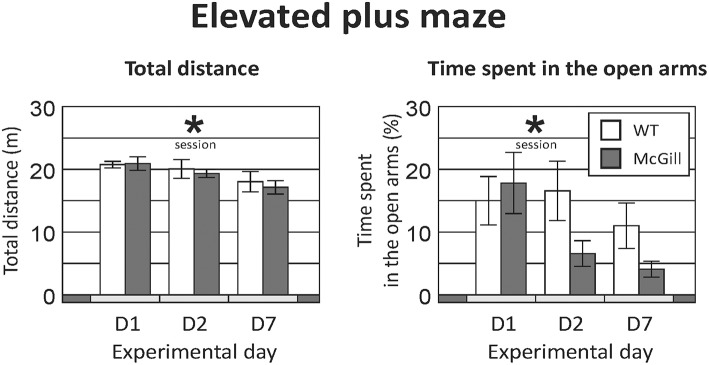
Elevated plus maze. Total distance **(Left)** significantly decreased over time (*p* < 0.01), suggesting a decline of exploratory activity with subsequent exposures, but without any effect of genotype or interaction. Time spent in the open arms **(Right)** also decreased significantly in subsequent sessions (*p* < 0.01), without a significant effect of genotype. A trend-level (*p* = 0.06) interaction between genotype and session suggested a more pronounced decline in the homozygous McGill group. *p < 0.05.

**Total distance** walked by the animals exploring the maze was not affected by their genotype, but it significantly decreased across subsequent sessions (Figure 1; Table 4), suggesting gradual habituation and decreasing exploratory activity in both groups.

**Table 4:** ANOVA summary table for elevated plus maze.

**Source**	**df**	**df within group**	***F***	***p***
**TOTAL DISTANCE**				
Session	2	36	8.117	0.0012^**^
Genotype	1	36	0.1445	0.71
Session x genotype	2	36	0.2439	0.78
**FRACTION OF TIME SPENT IN THE OPEN ARMS**				
Session	2	36	5.25	0.01^**^
Genotype	1	36	1.321	0.265
Session x genotype	2	36	2.983	0.0633

*All differences above trend level (p < 0.1) are in bold*.

** *p < 0.01*.

The **fraction of time spent in the open arms** also decreased over time while there was no effect of group and a trend-level interaction, suggesting a faster change in the McGill group (Figure 1; Table 4). In the McGill group, balance problems occurred in the open arms, with three individuals actually falling down from the apparatus.

### Open field test

**Figure 2:**
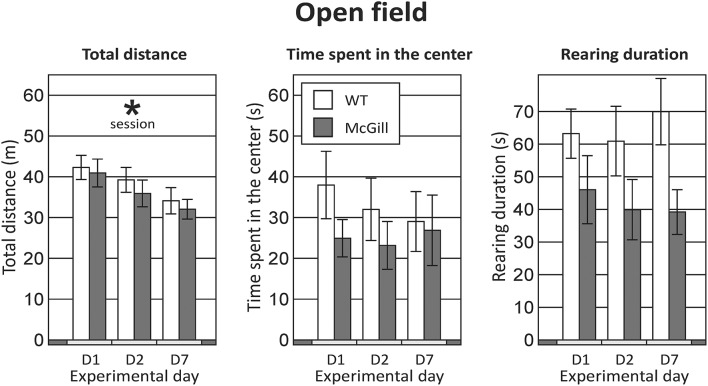
Open field. Behavior in the OF was not significantly affected by the genotype of the animals in any of the parameters assessed (the difference in rearing duration failed to reach significance). Repeated exposures significantly decreased locomotor activity (*p* < 0.001; left panel), without an interaction with genotype. *p < 0.05.

**Total distance** walked in the OF was not affected by genotype, but significantly decreased with repeated sessions (Figure 2; Table 5).

**Table 5:** ANOVA summary table for open field.

**Source**	**df**	**df within group**	***F***	***p***
**TOTAL DISTANCE**				
Session	2	36	10.39	0.0003^***^
Genotype	1	36	0.359	0.56
Session x genotype	2	36	0.1424	0.87
**TIME SPENT IN THE CENTER**				
Session	2	36	0.2205	0.80
Genotype	1	36	1.154	0.30
Session x genotype	2	36	0.3971	0.68
**TIME SPENT BY REARING**				
Session	2	36	0.8280	0.45
Genotype	1	36	3.451	**0.0797**
Session x genotype	2	36	1.747	0.19

*All differences above trend level (p < 0.1) are in bold*.

*** *p < 0.001*.

Animals from both groups spent most time in the periphery and rarely visited **the central part** of the maze (Figure 2; Table 5).

Time spent by **rearing** was slightly but non-significantly lower in the McGill rats. For other behaviors (grooming, freezing, defecation) no effects were apparent (not shown).

### Beam walking

Most of the animals were motivated to solve the task by crossing the beam and reaching the goal box with cage mates. Two individuals from the McGill group had to be excluded; one because of an injury, the other because of excessive anxiety and immobility when placed on the beam. Some animals were unable to walk the narrow beam, and dragged their belly on the upper surface instead, hugging the beam with their hind limbs. With the criteria we used, it was impossible to count foot slips in those cases, as those animals “slipped” all the time. This was counted as the maximum score of 10 foot slips.

**Figure 3:**
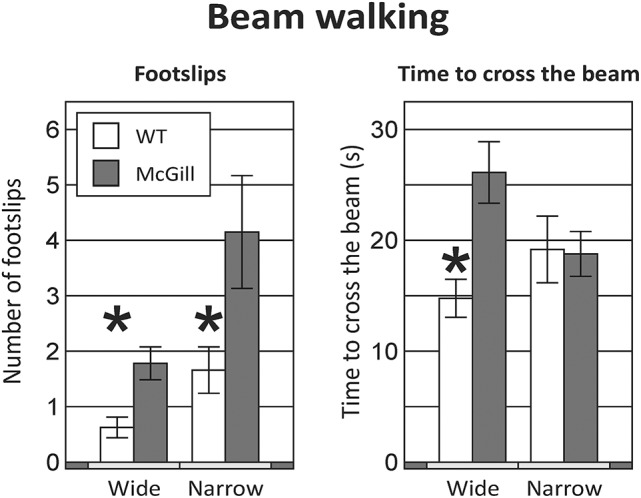
Beam walking. Animals of the McGill group exhibited a higher number of foot slips when walking on either the wide or narrow beam **(Left)**, and were significantly slower on the wide beam, but not the narrow beam **(Right)**. *p < 0.05.

McGill rats exhibited more foot slips on both the wide beam and the narrow beam (Figure 3; Table 6). Moreover, other signs of impaired locomotor abilities (falls, near-falls or belly dragging) occurred in this group while being almost absent among the controls. However, the number of those behaviors was not sufficient for meaningful statistical evaluation, so we report them only in the form of a table (Table 7). McGill rats also more often exhibited signs of distress during the experiments.

**Table 6:** T-test summary table for beam walking.

**Source**	**df**	**df within group**	***T***	***p***
**Beam walking**				
Foot slips—wide beam	1	16	3.429	0.0034^**^
Foot slips—narrow beam	1	16	2.445	0.0264^*^
Time to cross—wide beam	1	16	3.416	0.0035^**^
Time to cross—narrow beam	1	16	0.100	0.92

*All differences above trend level (p < 0.1) are in bold*.

* *p < 0.05*;

** *p < 0.01*.

**Table 7:** Beam walking test results.

**Event**	**Near-fall (wide)**	**Near-fall (narrow)**	**Fall (wide)**	**Fall (narrow)**	**Belly-dragging (narrow)**
Number McGill	23	9	8	1	7
Number WT	9	2	1	1	0

McGill rats required more time to cross the beam in the initial wide configuration, but not in the narrow beam sessions (Figure 3; Table 6).

### Y-maze

**Figure 4:**
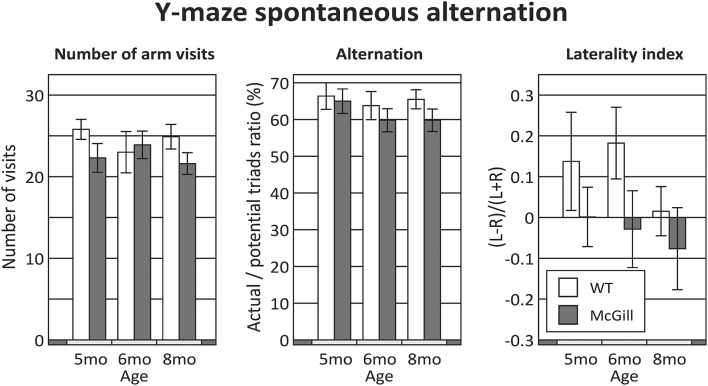
Y-maze. Behavior in the Y-maze was not significantly affected by either the group, age or their interactions in any of the evaluated parameters. The difference in the laterality index (with 5 and 6 month old WT animals seemingly preferring left turns over right turns) was not significant.

The McGill rats did not differ from controls in any of the evaluated parameters (Figure 4; Table 8). Both groups slightly preferred alternation over random behavior, as their score was above the expected random value (50%), and they did not use a lateralized behavioral strategy (e.g., turning always left or always right).

**Table 8:** ANOVA summary table for the Y-maze.

**Source**	**df**	**df within group**	***F***	***p***
**NUMBER OF ARM VISITS**				
Session	2	36	0.1543	0.86
Genotype	1	36	1.058	0.32
Session x genotype	2	36	1.374	0.27
**RATIO OF ACTUAL TO POTENTIAL TRIADS**				
Session	2	36	0.6331	0.54
Genotype	1	36	2.210	0.15
Session x genotype	2	36	0.1717	0.84
**LATERALITY INDEX**				
Session	2	36	1.339	0.27
Genotype	1	36	1.880	0.19
Session x genotype	2	36	0.3378	0.72

### Morris water maze

**Figure 5:**
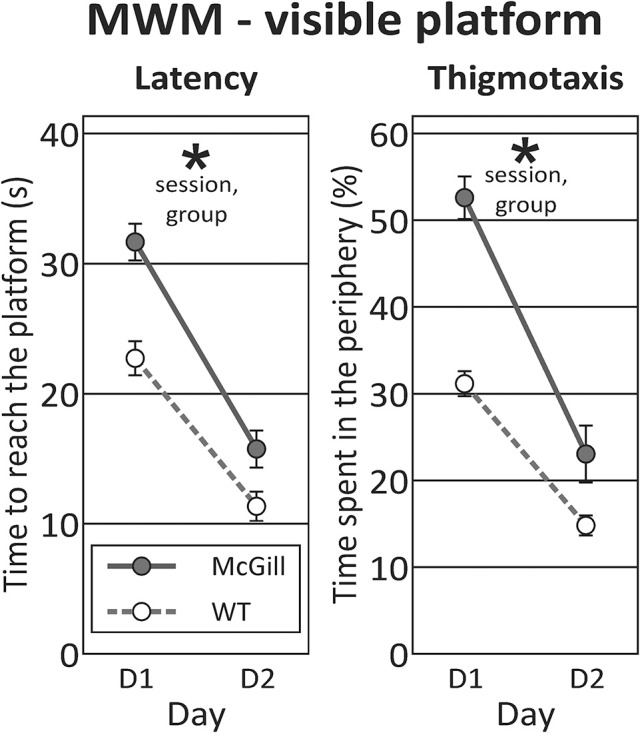
Morris water maze - visible platform task. In visible platform testing, both groups improved their latency to find the platform (*p* < 0.0001; left panel), with McGill rats performing significantly worse (*p* < 0.001) than WT controls. Their poor performance was linked to persistent thigmotaxis. McGill rats spent more time in the pool periphery (*p* < 0.0001). While thigmotaxis decreased with experience (effect of session, *p* < 0.0001), this trend was more pronounced in the McGill group (interaction between session and genotype, *p* = 0.01). *p < 0.05.

In the **visible platform** task, McGill rats exhibited significantly longer latency to reach the platform than the controls, significantly improved across the sessions, and there was a trend toward interaction between group and session. McGill group also exhibited elevated thigmotaxis, which was more pronounced in the first session compared to the second one, and there was a significant interaction between group and session (Figure 5; Table 9).

**Table 9:** ANOVA and *t*-test summary table for the Morris water maze.

**Source**	**df**	**df within group**	***F***	***p***
**VISIBLE PLATFORM—LATENCY**				
Session	1	18	146	<0.0001^***^
Genotype	1	18	17.1	0.0006^***^
Session x genotype	1	18	4.03	**0.0601**
**VISIBLE PLATFORM—THIGMOTAXIS**				
Session	1	18	41.8	<0.0001^***^
Genotype	1	18	87.6	<0.0001^***^
Session x genotype	1	18	8.28	0.01^*^
**HIDDEN PLATFORM—LATENCY**				
Trial	3	54	18.73	<0.0001^***^
Genotype	1	18	40.59	<0.0001^***^
Session x genotype	3	54	0.9676	0.41
**HIDDEN PLATFORM—THIGMOTAXIS**				
Trial	3	54	5.919	0.0014^**^
Genotype	1	18	24.29	0.0001^***^
Session x genotype	3	54	1.593	0.2018
**Source**	**df**	**df within group**	***T***	***p***
**HIDDEN PLATFORM—SAVINGS OF TIME**				
Short delay	1	18	0.1154	0.91
Long delay	1	18	1.788	**0.0907**
**HIDDEN PLATFORM—SWIMMING SPEED**				
Speed	1	18	1.463	0.1607

*All differences above trend level (p < 0.1) are in bold*.

* *p < 0.05*;

** *p < 0.01*;

*** *p < 0.001*.

The visual inspection of rat behavior showed that McGill rats exhibited no overt locomotor impairment, but were slower to adopt an efficient platform searching strategy, and less prone to swim directly toward the visual cue.

**Figure 6:**
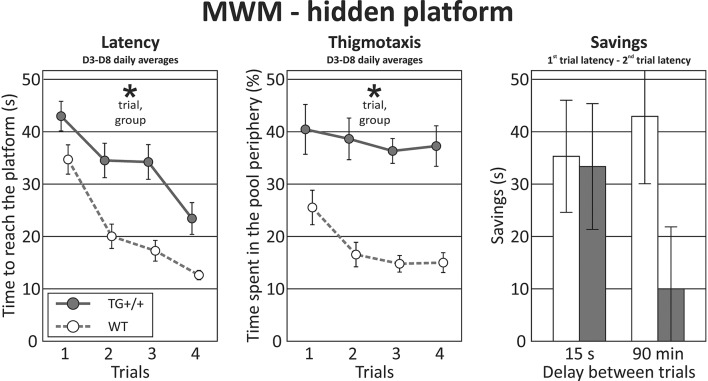
Morris water maze - hidden platform task. In the hidden platform version of the MWM, McGill rats exhibited longer latencies than their control counterparts (effect of group, *p* < 0.0001, left panel). The performance of both groups improved with subsequent trials (effect of trial, *p* < 0.0001), with no interaction between the factors. Thigmotaxis (middle panel) was again higher in the McGill group (*p* = 0.0001), but in both groups tended to decrease with subsequent trials (*p* = 0.0014). Savings (a measure of improvement between the first and the second trial within a day) were not significantly different, with a trend toward lower performance of the McGill group when the delay was longer (*p* < 0.1). *p < 0.05.

In the **hidden platform** task, the latency during individual trials (averaged across training days) was longer in the McGill group, and in both groups improved with subsequent trials. Thigmotaxis was again higher in the McGill rats and decreased with subsequent trials (Figure 6; Table 9).

The savings of time between the first and second trials of every session are a measure of single-trial learning. When the delay between the sessions was short, there was no effect of group. However, after a long (90-min) delay, there was a trend toward McGill animals performing worse (Figure 6; Table 9).

Swimming speed (all-session average for each animal) was the same in both groups, which shows that the effects were not due to locomotor impairment in the McGill group (Table 9).

### Carousel maze

Visual observations of the animals suggested that both McGill and controls were motivated to forage for groats, and also visibly reacted to the electric punishment, suggesting a normal pain threshold. Ultrasonic vocalizations around 22 kHz, while absent in the control rats, occurred in three individuals of the McGill group during the active place avoidance training. They could be linked to anxiety caused by their inability to master the task, but the number of vocalizing individuals was too low for statistical analysis.

In the three initial sessions of **spontaneous foraging**, McGill rats exhibited normal locomotor activity. The interaction between the factors of group and session reached the level of a trend, suggesting that locomotion in the McGill group gradually decreased while the controls became more active over time.

**Figure 7:**
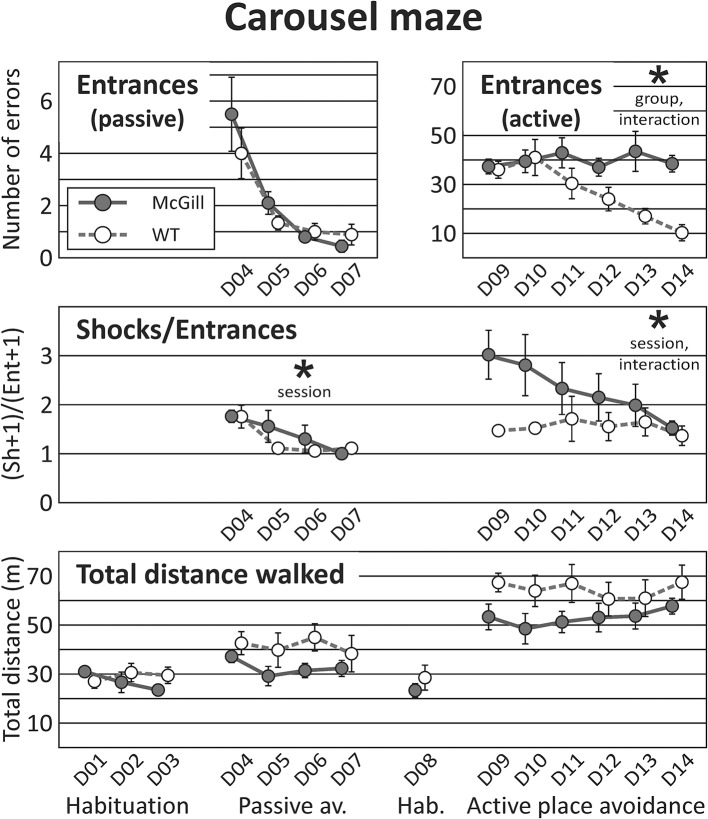
Carousel maze. Carousel maze testing consisted of habituation on a stable arena, 4-day passive avoidance training, one 10-min habituation session on the rotating arena, and 6-day active place avoidance training. The number of entrances into the to-be-avoided sector in the passive place avoidance (**top left** panel) decreased in subsequent sessions, and was not affected by genotype. In the active avoidance (**top right**, note different scale of the Y-axis), McGill rats made more entrances (effect of group, *p* < 0.05), and failed to improve with sessions (interaction between session and group, *p* < 0.005). The number of shocks per entrance (skill learning index, **middle** panel) did not differ between the groups in either task, but did improve over time for each particular task. In the active place avoidance, the McGill group was initially worse but managed to improve in this parameter (effect of interaction between session and group, *p* < 0.05). Total distance walked during a session (**bottom** panel) was similar in both groups. *p < 0.05.

In the **passive place avoidance** (but not in other stages of the task), McGill animals seemed to eat fewer groats than control subjects (visual observation). This could be a short-term effect of the introduction of shocks. After becoming familiarized with the task rules, all animals usually escaped from the to-be-avoided sector after the first shock, suggesting good motivation and locomotor response. Entrances into the to-be-avoided sector (avoidance failures) were not affected by the factor of group, but decreased in subsequent sessions, demonstrating gradual improvement in both groups. The number of shocks per entrance (skill learning index) did not differ between groups, and improved across sessions (Figure 7; Table 10).

**Table 10:** ANOVA and *t*-test summary table for the carousel maze.

**Source**	**df**	**df within group**	***F***	***p***
**SPONTANEOUS FORAGING—TOTAL DISTANCE**				
Session	2	36	0.8212	0.45
Genotype	1	18	0.2860	0.60
Session x genotype	2	36	2.86	**0.0704**
**PASSIVE PLACE AVOIDANCE—TOTAL DISTANCE**				
Session	3	51	1.91	0.14
Genotype	1	17	1.77	0.20
Session x genotype	3	51	1.35	0.27
**PASSIVE PLACE AVOIDANCE—ENTRANCES**				
Session	3	51	14.4	<0.0001^***^
Genotype	1	17	0.572	0.46
Session x genotype	3	51	0.820	0.49
**PASSIVE PLACE AVOIDANCE—SHOCKS PER ENTRANCE**				
Session	3	51	7.449	0.0003^***^
Genotype	1	17	0.4827	0.4966
Session x genotype	3	51	1.221	0.3114
**ACTIVE PLACE AVOIDANCE—TOTAL DISTANCE**				
Session	5	80	1.04	0.40
Genotype	1	16	2.29	0.15
Session x genotype	5	80	0.796	0.56
**ACTIVE PLACE AVOIDANCE—ENTRANCES**				
Session	5	80	3.38	0.0081^**^
Genotype	1	16	6.73	0.0195^*^
Session x genotype	5	80	3.86	0.0035^**^
**ACTIVE PLACE AVOIDANCE—SHOCKS PER ENTRANCE**				
Session	5	80	2.62	0.0302^*^
Genotype	1	16	2.08	0.1685
Session x genotype	5	80	2.35	0.0486^*^

*All differences above trend level (p < 0.1) are in bold*.

* *p < 0.05*;

** *p < 0.01*;

*** *p < 0.001*.

In the **active place avoidance**, the number of entrances was significantly higher in McGill rats, and the significant interaction between group and session showed that only control rats improved across the sessions. The number of shocks per entrance (skill learning index) did not differ between groups, but improved across sessions with a significant interaction of group and session factors (Figure 7; Table 10). While control rats exhibited good escape skills from the very beginning, McGill rats were slower to adopt the correct strategy (running out of the sector immediately after receiving a shock), although they eventually became as good as their healthy counterparts in this respect.

### Light-dark test

**Figure 8:**
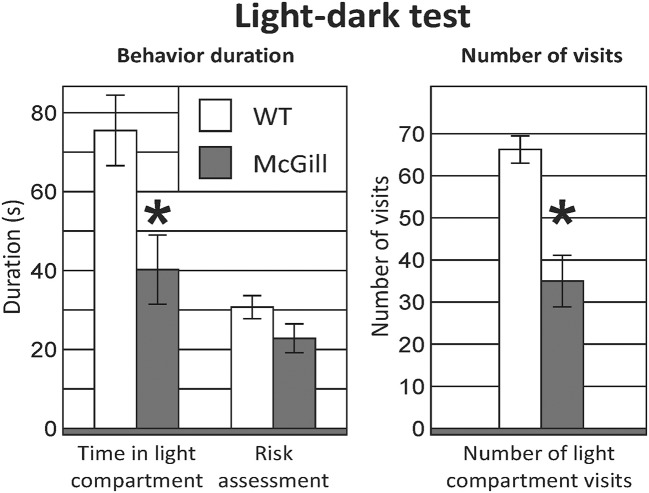
Light-dark test. McGill rats spent less time in the light compartment (*p* < 0.05) and entered it less often (*p* < 0.001). Risk assessment behavior (looking into the light compartment without entering) was not significantly decreased in McGill rats. *p < 0.05.

McGill rats spent significantly less time in the light compartment and entered it less often (Figure 8; Table 11).

**Table 11:** *T*-test summary table for the light-dark test.

**Source**	**df**	**df within group**	***T***	***p***
**Light-dark test**				
Time in the light compartment	1	18	2.813	0.0115^*^
Number of entrances	1	18	4.506	0.0003^***^
Risk assessment duration	1	18	1.690	0.11

*All differences above trend level (p < 0.1) are in bold*.

* *p < 0.05*;

*** *p < 0.001*.

### Social recognition task

**Figure 9:**
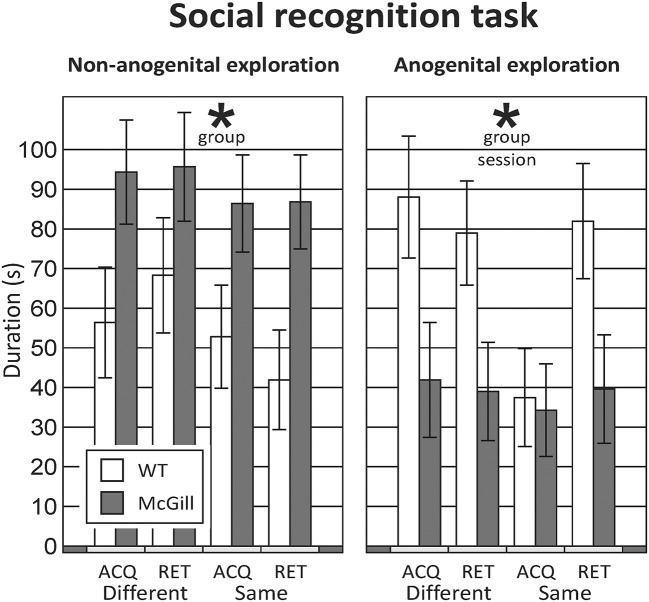
Social recognition task. The rats underwent four different sessions: in acquisition sessions (ACQ), they explored a novel stimulus animal, and in a retrieval session 30-min later were exposed either to a different animal (RET-Different) or to the familiar rat (RET-Same). Each rat was tested under both “Different” and “Same” conditions, in a randomized manner, to preclude biases caused by their order or time of day. In non-anogenital exploration (**left** panel), there was no effect of session, meaning that the behavior of the tested rat was not affected by the familiarity of the stimulus animal. In anogenital exploration (**right** panel), there was an effect of session, but it was not caused by a decrease of exploration in the “RET-Same” condition as hypothesized. The McGill rats in general performed significantly less anogenital exploration (*p* < 0.05), but more non-anogenital exploration (*p* < 0.01). *p < 0.05.

From the initial inspection of the data, it was clear that the task did not work as assumed, namely that even in the control subjects, non-anogenital exploration was not lower with a familiar relative to an unfamiliar stimulus animal, and anogenital exploration even increased with repeated exposure to the same rat; therefore the basic assumption of decreasing social investigation with repeated exposures was not met. Moreover, the behavior of the animals was not stable between testing days 1 and 2 (measures of social exploration evolved with subsequent exposures, regardless of familiarity). Therefore, it would not be correct to interpret the results as reflecting habituation to stimulus animals and no conclusions about social memory could be made. Therefore, we decided to focus simply on differences in social behaviors between groups, and analyzed the data by ANOVA for repeated measures. For **anogenital exploration**, there was a significant effect of genotype and session. With **non-anogenital exploration**, there was again a significant effect of genotype, but no effect of session (Figure 9; Table 12). In other behaviors, there were no significant differences between the groups (not shown).

**Table 12:** ANOVA summary table for the social recognition task.

**Source**	**df**	**df within group**	***F***	***p***
**ANOGENITAL EXPLORATION**				
Session	3	45	3.762	0.017^*^
Genotype	1	15	4.745	0.046^*^
Session x genotype	3	45	2.188	0.103
**NON-ANOGENITAL EXPLORATION**				
Session	3	45	0.736	0.536
Genotype	1	15	11.535	0.004^**^
Session x genotype	3	45	0.174	0.913

*All differences above trend level (p < 0.1) are in bold*.

* *p < 0.05*;

** *p < 0.01*.

### Social interaction with an unfamiliar adult

**Figure 10:**
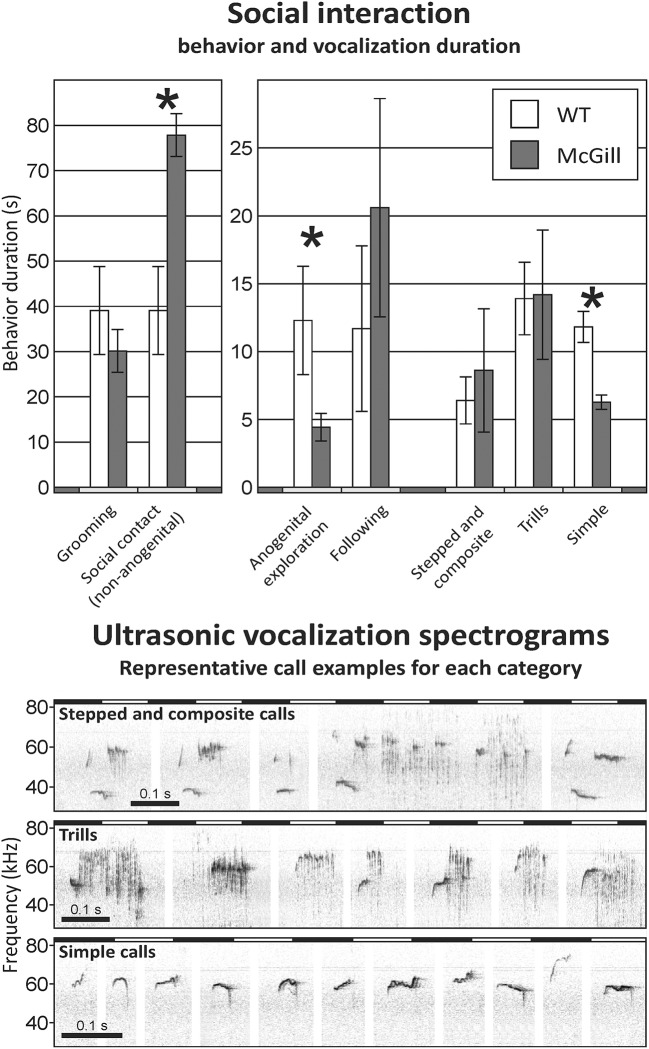
Social interaction. In social interaction settings, the McGill group spent significantly more time engaging in non-anogenital social contact (*p* < 0.001), while performing less anogenital exploration (*p* < 0.05). In following and self-grooming there were no significant differences. Simple high-frequency vocalizations were significantly less abundant in McGill group (*p* < 0.005), while the abundance of trills and stepped and composite vocalizations was similar. *p < 0.05.

McGill rats spent significantly more time engaged in non-anogenital social contact with the conspecifics, while performing less anogenital exploration. Simple high-frequency vocalizations were significantly less abundant in the McGill group (Figure 10; Table 13). Other vocalization subtypes did not differ between the groups. Low-frequency calls occurred rarely and therefore are not reported. No aggressive behavior was observed.

**Table 13:** *T*-test summary table for social interaction.

**Source**	**df**	**df within group**	***T***	***p***
**Social interaction**				
Anogenital exploration (duration)	1	18	2.556	0.0198^*^
Non-anogenital social contact (duration)	1	18	4.567	0.0002^***^
Following (duration)	1	18	1.188	0.2505
Grooming (duration)	1	18	1.111	0.2811
Simple high-frequency vocalization (number)	1	18	3.925	0.0044^**^
Stepped and composite vocalization (number)	1	18	0.4065	0.6695
Trills (number)	1	18	0.047	0.9637

*All differences above trend level (p < 0.1) are in bold*.

* *p < 0.05*;

** *p < 0.01*;

*** *p < 0.001*.

### Novel object location/recognition task

**Figure 11:**
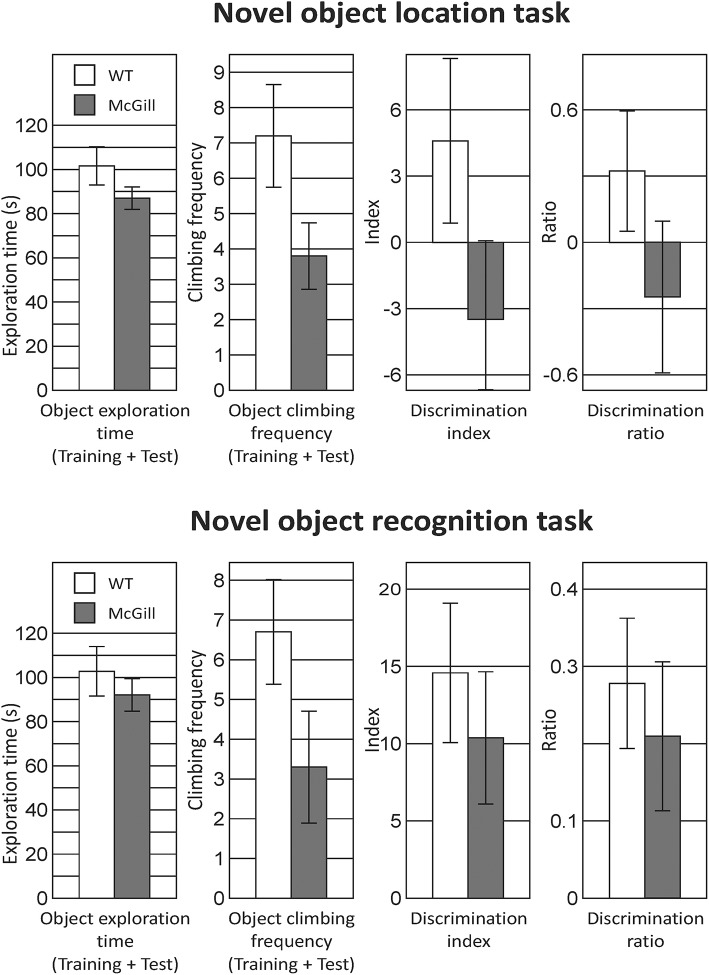
Novel object location and recognition task. In the twin tasks focused on recognition memory of object position or identity, there was no significant difference between McGill and control rats. General exploration behavior was similar, although McGill rats were less willing to climb onto the objects (a trend-level difference apparent in both tasks), presumably because of the balance problems noted in other tasks. In NOL, preference for a relocated object evidenced by the discrimination index and discrimination ratio was weak in both McGill and WT animals. In NOR, both groups preferred the novel object to the same extent.

In both tasks, object exploration was similar in both groups, but the control animals tended to climb onto the objects more often. In NOL, the preference for the relocated object was very low in both groups, while in NOR the animals clearly preferred the novel object, without any impairment in the McGill group (Figure 11; Table 14).

**Table 14:** *T*-test summary table for the novel object location/recognition task.

**Source**	**df**	**df within group**	***T***	***p***
**NOL/NOR**				
NOL—object exploration	1	18	1.459	0.1619
NOL—object climbing	1	18	1.966	**0.0649**
NOL—discrimination index	1	18	1.607	0.1255
NOL—discrimination ratio	1	18	1.304	0.2087
NOR—object exploration	1	18	0.7989	0.4348
NOR—object climbing	1	18	1.764	**0.0946**
NOR—discrimination index	1	18	0.6761	0.5076
NOR—discrimination ratio	1	18	0.5358	0.5986

*All differences above trend level (p < 0.1) are in bold*.

### Circadian locomotor activity cycle

**Figure 12:**
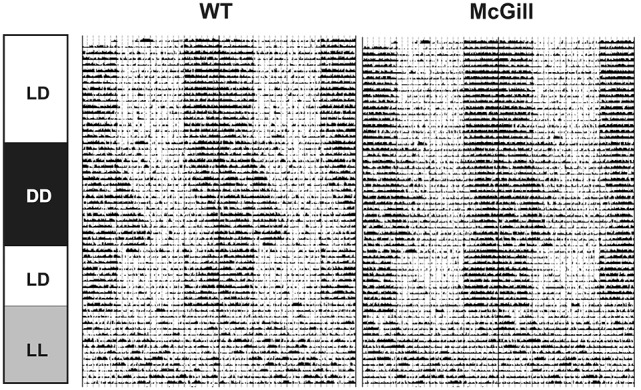
Representative double-plotted actograms of spontaneous locomotor activity of a single control WT **(Left)** and McGill rat **(Right)**. Boxes on the left indicate lighting protocols used during the recording (12 h light and 12 h dark, LD; constant darkness, DD; constant light, LL).

**Figure 13:**
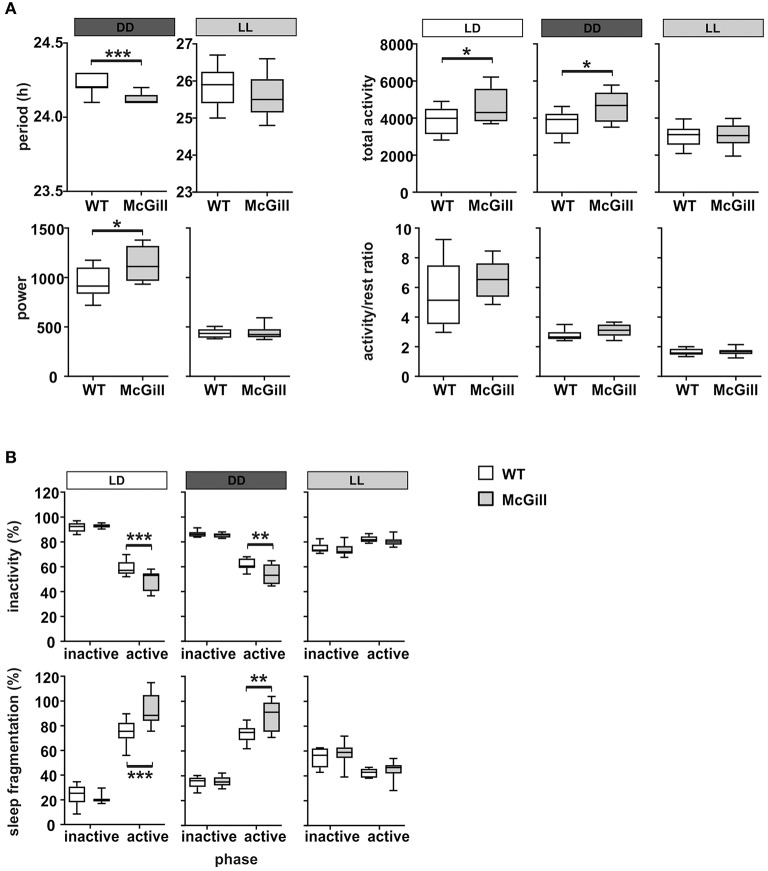
Analysis of locomotor activity parameters. **(A)** Period, estimation of the power of period, total activity and the activity/rest ratio of WT (empty boxes) and McGill (gray boxes) rats analyzed in LD (white bar above the plot), DD (dark gray bar), or LL (gray bar), respectively. **(B)** Inactivity (percentage of time spent resting) and the sleep fragmentation index (percentage of restlessness during inactivity) of WT and McGill rats analyzed separately for inactive (i.e., daytime on LD) and active (i.e., nighttime on LD) periods during LD, DD, and LL regimes. The data are expressed as median with 25 to 75th percentile box and min to max value whiskers, *n* = 9–10/group. The results of the statistical comparisons between groups are depicted. *p < 0.05; **p < 0.01; ***p < 0.001.

To characterize their circadian behavior, the spontaneous locomotor activity of McGill and control rats was monitored throughout the protocol. The activity records (double-plotted actograms) of a single representative McGill and WT rat are shown in Figure 12. The analysis of the locomotor activity of all animals (Figure 13A; Table 15) revealed that under LD the activity exhibited a clear daily rhythm, with increased activity levels during the dark phase and decreased levels during the light phase in both rat genotypes. Activity profiles revealed no apparent phase difference between the strains. The total activity was slightly higher in McGill compared to controls in LD and DD, but not in LL conditions. The amplitude of the rhythms assessed as the activity/rest ratio was not different between the groups under any of the tested conditions. The period of the circadian clock in the SCN was assessed according to periodograms of free-running locomotor activity rhythm in constant conditions. In DD, the period was significantly shorter in McGill rats compared to controls and the rhythm had significantly higher power. In LL neither the periods nor the power differed between the groups. In order to analyze the behavioral parameters in more detail, we calculated the fraction of time that the animals spent inactive (immobile) and the index of inactivity fragmentation separately during the active and inactive phase of the circadian cycle (Figure 13B). The results showed that McGill rats had lower % of inactivity intervals during the active phase in LD and DD, but not in LL. Moreover, the inactivity bouts during the active phase were more fragmented in McGill rats under LD as well as DD. The data revealed that the slightly higher total activity during 24 h interval found in McGill rats was not due to a disturbance of their sleep but rather due to their restlessness during the active phase, during which they showed fewer and more fragmented bouts of idleness.

**Table 15:** *T*-test summary table for circadian activity.

**Source**	**df**	**df within group**	***T***	***p***
**Circadian activity**				
Total activity—LD	1	17	2.178	0.044^*^
Total activity—DD	1	17	2.425	0.0267^*^
Locomotor activity period—DD	1	15.61	4.159	0.0008^***^
Locomotor activity rhythm power—DD	1	17	2.372	0.0298^*^
Fraction of time immobile in active phase—LD	1	34	4.336	0.0002^***^
Fraction of time immobile in active phase—DD	1	34	3.376	0.0037^**^
Inactivity fragmentation—LD	1	34	4.233	0.0003^***^
Inactivity fragmentation—DD	1	34	3.803	0.0011^**^

*All differences above trend level (p < 0.1) are in bold*.

* *p < 0.05*;

** *p < 0.01*;

*** *p < 0.001*.

### Clock gene expression in the brain

**Figure 14:**
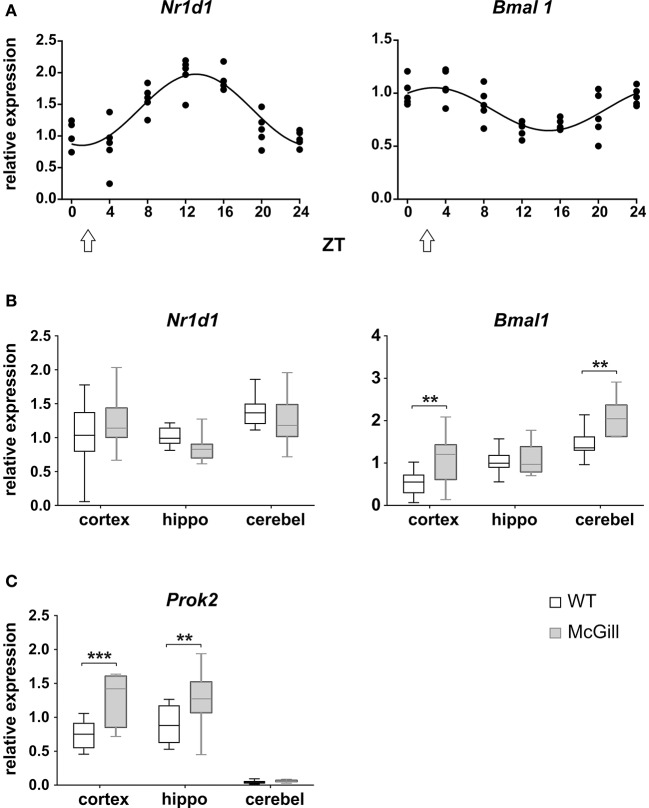
Analysis of mRNA levels. **(A)** Circadian profile of *Nr1d1* and *Bmal1* gene expression in the cerebellum of WT rats. The rats were sacrificed in 4-h intervals during 24 h. Time is expressed as *Zeitgeber* time (ZT); ZT0 represents the time of lights on in the previous LD12:12 cycle. The data are fitted with a cosinor curve; *n* = 4–5/time point. **(B,C)** Relative expression of *Nr1d1* and *Bmal1* **(B)** and *Prok2* **(C)** in the cortex, hippocampus (hippo) and cerebellum (cerebel) of experimental control WT (white) and McGill (gray) rats. The animals were sacrificed at ZT3 (as depicted by the white arrow in **A**). The data are expressed as median with 25 to 75th percentile box and min to max value whiskers, *n* = 9–10/group. The results of the statistical comparisons between groups are depicted. **p < 0.01; ***p < 0.001.

The functional state of the circadian clocks was assessed in three brain areas, namely in the hippocampus, parietal cortex and cerebellum. First, we analyzed the full daily profile of *Nr1d1* and *Bmal1* expression by RT qPCR in the cerebellum of a separate group of Wistar rats (Wistar:Han, bred at the Institute of Physiology, CAS, *n* = 35) that were maintained in the same light/dark conditions as the experimental groups and then sacrificed in 4 h intervals during the 24 h daily cycle (Figure 14A): the expression of the clock genes *Nr1d1* and *Bmal1* exhibited significant circadian rhythms and their phases were opposite to each other, as expected based on the current model of the molecular clock. The results provided information about the expected phase of the clock in the brain areas studied in the WT controls (*n* = 10) and McGill rats (*n* = 10). Experimental animals were sacrificed at a single time point (depicted by an arrow in Figure 14A) during the daytime, i.e., 3–4 h after lights-on (*Zeitgeber* time 3–4), which is the time point when the *Nr1d1* expression profile was lowest and the *Bmal1* expression profile was at its peak. The results revealed differences in clock gene expression between the genotypes (Figure 14B; Table 16), with *Bmal1* expression in McGill rats being significantly higher in the parietal cortex and cerebellum when compared to controls. The differences between the groups were not significant for *Nr1d1* expression. Moreover, we analyzed the expression of the clock-controlled gene *Prok2* (Figure 14C), which codes for a chemokine neuropeptide suspected to be a mediator of brain-damage and is upregulated by various stressors including hypoxia and inflammation (Cheng et al., 2012). *Prok2* mRNA levels were significantly higher in the parietal cortex as well as in the hippocampus of McGill rats when compared to controls. Its levels were below the level of detection in the cerebellum of both genotypes. Overall, the data suggest that the functional state of the circadian clocks in these brain areas might differ between these genotypes.

**Table 16:** *T*-test summary table for clock gene expression.

**Source**	**df**	**df within group**	***T***	***P***
**Clock gene expression**
*Bmal1*—parietal cortex	1	53	3.14	0.0055^**^
*Bmal1*—cerebellum	1	53	3.401	0.0038^**^
*Prok2*—parietal cortex	1	52	4.452	0.0001^***^
*Prok2*—hippocampus	1	52	3.29	0.0036^**^

*All differences above trend level (p < 0.1) are in bold*.

** *p < 0.01*;

*** *p < 0.001*.

## Discussion

Our results show that homozygous McGill rats exhibit a complex phenotype and behavioral impairments beyond those already described in the literature.

Impaired spatial cognition is the hallmark of AD (Vlcek, 2011) and its presence in the McGill rats is well-documented in the literature (Leon et al., 2010). In the MWM, we observed significantly worse performance in the hidden platform task as expected, and a hint of specifically impaired long-term retention of spatial information. The deficit was not limited to spatial memory, however, because the animals also performed worse in the visible platform (cued) trials, and exhibited persisting thigmotaxis with both a hidden and visible platform, despite no indication of locomotor problems. We have to note that impaired spatial cognition *per se* does not prevent animals from abandoning thigmotactic behavior and developing adaptive non-spatial strategies, such as chaining or selective scanning of the central part of the maze, which was well-described in a mouse model of AD by Janus (2004), but did not occur in our McGill group. The findings therefore suggest a more generalized behavioral impairment, reaching beyond the cognitive domain. In this aspect our results differ from the work of Leon et al. (2010), who found no deficit in visible platform trials even in 13 month old homozygous animals and reported purely cognitive impairment.

In the carousel maze, McGill rats performed quite well in the passive avoidance paradigm, which has low demands on spatial navigation. In the active place avoidance the McGill rats were evidently unable to manage information from dissociated reference frames for successful avoidance, as documented by their persistently high number of entrances, while the controls steadily improved. What the McGill rats gradually managed to learn was to escape immediately after receiving a shock, and thus minimize the time spent in the to-be-avoided sector. Thus, they demonstrated the ability to adopt an adaptive, although non-spatial strategy (in contrast to their water maze behavior). This impairment of spatial cognition is suggestive of a severe deficit of hippocampal function, which fits the symptoms observed in human patients with AD, or people with amnestic MCI preceding the actual onset of dementia (Gazova et al., 2015). In the McGill model, the hippocampal formation is likewise among the first structures affected (Leon et al., 2010).

The spontaneous alternation in the Y-maze paradigm has been previously investigated only in *hemizygous* McGill rats by Galeano et al. (2014) and Martino Adami et al. (2017a,b), who found a significant deficit of spontaneous alternation. The discrepancy with our results is puzzling, because the experimental design and age of the animals were similar, and the homozygous animals employed in the present study should have exhibited a more severe phenotype.

Recognition memory for object identity and position in the NOR and NOL tasks were already employed in McGill rats by Iulita et al. (2014), who found profound impairments of recognition memory in both tasks at the ages of 3 and 13 months in both hemi- and homozygous animals, quite unlike our findings. However, their paradigm (using five objects and a short, 10-min delay between sampling and test session) was not directly comparable to our study here. Galeano et al. (2014) and Martino Adami et al. (2017a,b) found no impairment in hemizygous McGill rats using a NOR design similar to ours.

Exploratory behavior and locomotor activity were normal in the McGill rats in the OF and the EPM and showed normal habituation with repeated exposures, providing indirect evidence of the ability to recognize a familiar environment, even after a 7-day delay. The decrease in open arm exploration in the EPM seemed rather more pronounced in the McGill rats (although the difference failed to reach significance). This observation may be partially explained by the McGill animals having balance problems and avoiding the open arms due to their substantiated fear of falling down. Galeano et al. (2014) and Martino Adami et al. (2017a,b) reported normal locomotor behavior in EPM and OF, which is in line with our results. However, they found increased anxiety in the OF only (thigmotaxis). In our experiment, rats from both groups hardly ever visited the central area, possibly masking the effect of genotype in this task. Elevated anxiety in the McGill group was shown by our data from the light-dark test, and might have affected behaviors observed in the MWM (increased thigmotaxis) and the carousel maze (anxious vocalization). Anxiety is a common occurrence in animal models of AD (e.g., Lee et al., 2004; Pentkowski et al., 2018, and references therein) as well as in elderly humans suffering from MCI or AD (Porter et al., 2003; Beaudreau and O'hara, 2008).

We confirmed the suspected problems with gait and balance in the beam walking test, where McGill rats exhibited an increased incidence of foot slips and falls. Motor impairments have not been tested in previous studies on McGill rats, with the exception of swimming abilities, which have been found to be normal by both Leon et al. (2010) and the present work. However, motor coordination impairment was reported in an analogous mouse model bearing APP with Swedish and Indiana mutations (Lee et al., 2004). Gait problems are frequently observed in AD patients (Visser, 1983; Suttanon et al., 2012).

We hypothesized that in the two tasks featuring interaction with a conspecific, McGill rats would exhibit impaired social memory and general social withdrawal. We could not test the former due to the reasons described in section Social Recognition Task, and the total time of social interactions was not notably decreased. However, we found a qualitative difference in the social behavior of McGill rats. In two different social contexts, they exhibited increased non-anogenital and decreased anogenital exploration, accompanied with a different profile of ultrasonic vocalizations. We cannot offer a definitive explanation for the different behavioral profile, but it should be noted that anogenital sniffing and licking is the major source of information enabling individual recognition in rats (Moura et al., 2010). We therefore hypothesize that the McGill rats were less motivated to establish the identity of their social partners, although otherwise willing to interact with them. Impaired olfaction, preventing the perception of identity-related cues, could be a plausible explanation, as impaired sense of smell has been reported in human AD and MCI patients (Devanand et al., 2000; Field, 2015) and mouse AD models (Wesson et al., 2010). We cannot test this possibility with the available data, but it would be a promising direction for future experiments.

We found that the circadian pattern of home-cage activity was comparable in both groups; however, McGill rats had a shorter free-running period and were slightly more mobile during their active phase (subjective night). During the day, their activity was similar to controls, which fits well with the results from the open field and plus maze tests. On the other hand, Wilson et al. (2017) found decreased home-cage activity in the McGill rat model, but they monitored the animals for a much shorter period of time (2 h) and did not specify the time at which the activity was measured, making their results difficult to compare with ours.

Multiple studies have described circadian locomotor activity specifically in transgenic AD models of mice (Chauhan et al., 2017). APPxPS1 mice (Duncan et al., 2012) showed only modest effects on sleep-wake rhythms, but the analysis was complicated by the prominent daytime activity peak of the background mouse strain, which is not present in the Wistar rats used as a background for the McGill rat model. APPxPS1 mice (Song et al., 2015) were also reported to have markedly decreased activity during the active night phase but not during the day, as well as increased sleep fragmentation, unlike McGill rats. Male 3xTg-AD mice (Sterniczuk et al., 2010) were reported to show a shorter free-running period and increased activity, similar to our data in McGill rats. Unlike McGill rats, however, they also showed less nighttime (active phase) activity. TgCRND8 mice showed modified activity patterns and reduced total activity (Ambrée et al., 2006), in contrast to McGill rats. In Tg2576 mice at all ages studied, the circadian period of wheel running rhythms in constant darkness was significantly longer than that of wild type mice (Wisor et al., 2005), again in contrast to McGill rats. CRND8 mice showed significantly more frequent, but shorter, bouts of wheel-running activity (Graybeal et al., 2015). Overall, the circadian activity of various mice transgenic AD models seems to be highly strain-dependent, though some of the variance could be attributed to different methodologies (e.g., camera recording or wheel running activity instead of spontaneous locomotion). Transgenic rat models of AD were developed later than mouse models, and to our knowledge this is the first report on their circadian characteristics. Interestingly, rats with Abeta expressing cells grafted to their SCNs showed high levels of activity during the light (inactive) phase and disrupted rhythms in activity and body temperature (Tate et al., 1992).

Previous research on human postmortem AD brains has revealed neuronal cell loss and reduced vasopressin levels in the SCN (Swaab et al., 1985; Hofman and Swaab, 1994; Stopa et al., 1999), and there is *in vitro* evidence for severe disruption of the circadian clock by the presence of Abeta (Schmitt et al., 2017), though the extent of clock gene expression changes in the SCN of AD patients is unknown. Output pathways projecting from the SCN to other parts of the brain could also be affected by AD progression and would play a major role in disrupting the circadian system. A study on human postmortem brains revealed changes in the phasing of circadian gene expression in other parts of the brain (Cermakian et al., 2011). However, our previous results showed only moderate changes in melatonin levels and peripheral clock gene expression in AD patients in their home environment (Weissová et al., 2016). So far, there is little evidence of disrupted clock gene expression in transgenic mouse and rat models of AD or their role in the neurodegenerative process, due also to the variety of phenotypes displayed by these models. Previous reports on APPxPS1 mice (Duncan et al., 2012; Song et al., 2015) found increased degradation of BMAL1 protein accompanied by increased *Bmal1* and decreased *Per2* mRNA amplitude in the SCN. Interestingly, we also found increased levels of *Bmal1* mRNA during its peak expression in the cortex and cerebellum of McGill rats. We found no evidence of decreased levels of the analyzed clock genes in the McGill brains areas due to the accumulation of Abeta.

Prokineticin 2 oscillates with high amplitude in the SCN and has been postulated as being one of the potential SCN output signals. It plays a physiological role in the circadian and homeostatic regulation of sleep (Hu et al., 2007), but also mediates and exacerbates proinflammatory states (Franchi et al., 2017). Interestingly, a recent report showed that *Prok2* mRNA is upregulated by Abeta treatment in the rat cortex and hippocampus, and *Prok2* antagonist protects the brain from Abeta-mediated neurotoxicity (Severini et al., 2015). The higher levels of Prok2 mRNA in the McGill cortex and hippocampus might also be due to a general increase of proinflammatory markers as was previously reported in the hippocampus of McGill rats (Hanzel et al., 2014). Finally, the higher *Prok2* mRNA levels correlate with observed increased activity of McGill rats, because prokineticin 2 plays a role in maintaining the awake state (Hu et al., 2007).

Overall, despite the significant differences in *Bmal1* and *Prok2* mRNA levels between the strains at the same time point, our data allow only for a limited interpretation. The number of available McGill rats precluded us from analyzing detailed time dynamics in gene expression, and we are thus unable to discuss e.g., possible differences in phase or amplitude.

Finally, we must mention some limitations of the McGill model and the present study. Firstly, the model has limited face validity, as some crucial aspects of AD, most notably the neurofibrillary tangles and the underlying tauopathy, are not reproduced. Secondly, the model is most applicable to early onset familial AD, as it has both a common cause (APP gene mutation) and similar outcome (Abeta accumulation and cognitive decline starting early in life). However, the predictive validity toward the predominant sporadic form of AD must be taken with caution.

The experiments presented in our study were conducted on a single group of age-matched rats. On the one hand, this eliminates some confounding factors such as batch effects and provides a complex multi-domain overview of the behavior, but on the other hand raises the issue of possible influence of repeated exposure to different tasks. Stress, which may be induced by the testing procedures, is known to influence behavior, especially learning, and may mask some cognitive impairments (Grootendorst et al., 2002). We avoided performing more than one behavioral task within a single day and allowed at least 2 days of rest after the more stressing tasks to prevent acute stress or fatigue from one test from directly affecting the performance in another, but this might not be sufficient to exclude all effects of repeated testing. For example, the exposure to multiple tasks and environments could be considered a form of environmental enrichment, which is known to alleviate the symptoms in AD models (Görtz et al., 2008).

The emotional, motor and social alterations observed in the present study require follow-up work to elucidate to what extent they can be considered analogous to the corresponding symptoms in human AD patients, and whether they could make the model useful in future studies of therapeutic approaches targeted to non-cognitive impairments.

## Conclusions

Our results confirm that the McGill transgenic rat model of AD exhibits good face validity at the behavioral level. The model exhibits postural or locomotor problems and increased anxiety. Social behavior and vocal communication are also altered in the model, but only in a qualitative, not quantitative manner, without signs of social withdrawal. We analyzed the circadian system of McGill rats for the first time and show evidence for moderate changes in circadian locomotor parameters as well as the functional state of the molecular clockwork in the brain of this AD model. Furthermore, we have confirmed that McGill rats display severe impairments of spatial cognition in both the classical Morris water maze task and the carousel maze testing battery, with less pronounced deficit in non-spatial tasks.

## Author contributions

TP was responsible for most of the behavioral testing and wrote the majority of the manuscript. IV conducted and evaluated the NOR and NOL experiments and participated in other behavioral tasks and their evaluation. VL and AP participated in behavioral testing of the animals and video data evaluation. MJ helped with the analysis of behavioral data from social interactions. HB helped with statistical evaluations. PH and MS were responsible for the experiments on the circadian system of the animal model. ASu, ZK, KV and ASt were responsible for scientific leadership and coordination of the study.

### Conflict of interest statement

The authors declare that the research was conducted in the absence of any commercial or financial relationships that could be construed as a potential conflict of interest.

## Abbreviations

Abeta: amyloid beta
AD: Alzheimer's disease
ANOVA: Analysis of variance
APP: amyloid precursor protein
CT: circadian time
DD: permanent darkness (condition in circadian testing)
EPM: elevated plus maze
ITI: inter-trial interval
LD: light/dark cycle (condition in circadian testing)
LL: permanent light (condition in circadian testing)
MCI: mild cognitive impairment
MWM: morris water maze
NOL: novel object location
NOR: novel object recognition
OF: open field
RT qPCR: quantitative reverse transcription polymerase chain reaction
SCN: suprachiasmatic nucleus
WT: wild type (non-transgenic)
ZT: *Zeitgeber* time.
